# Supplementary material for: Computational network biology analysis revealed COVID-19 severity markers: Molecular interplay between HLA-II with CIITA
Source: PLoS One. 2025 Mar 31;20(3):e0319205. doi: 10.1371/journal.pone.0319205 (PMC11957389; doi:10.1371/journal.pone.0319205)
Supplement: Attachment — Submitted filename: Response to Editor_R1.pdf [file pone.0319205.s001.pdf]

Response to Editor on

"Computational network biology analysis revealed COVID-19 severity markers: Molecular interplay between HLA-2 with CIITA"

We would like to thank you for insightful comments and suggestions. We carefully incorporated the comments and suggestions in the revised version of our manuscript. Our point by point responses are given in the following:

Efforts to set up new experimental approaches to gain new evidence that could enhance our understanding of the mechanisms underpinning the severe outcome of COVID-19 are valuable. The authors aimed to tackle the challenge through computational analysis of biological networks, developing a new strategy DGN detector on severe and non-severe samples of COVID-19. Regarding this point, there is a wide discrepancy in the sample size.

Could the authors explain this choice and if it might raise biases on the reliability of their statistical analyses?

Thank you for your comment, we agree with your comment.

We have described the limitation and related future work of our study in Discussion section as follow,

"In Section of COVID-19 severity specific gene network identification, we perform gene networks analysis for 368 sever and 105 non-severe samples. The analysis of the different sample sizes of phenotypes can be considered as one of limitations of our studies, because the different sample sizes may raise biases on the reliability. To avoid the bias from the different sample sizes, bootstrap strategy or randomly selected samples from 368 sever samples -based analysis can be considered as another future works of our studies." On page 12.

At the end of the introduction, the authors should summarize the results and briefly outline their meanings.

We have summarized our results in the end of Introduction Section as follows,

"The DRGdetector succeeded in revealing 15 differentially regulated gene networks of the markers. It can be through our results that the networks of severe samples showed dense gene networks compared with those of the non-severe samples. The identified one of the largest networks consists of the interplays between HLA class II gene family, i.e., HLA class II dominated the identified

differentially regulated gene network in COVID-19 severe samples, while the interplays became weaker and/or disappeared in that of non-severe samples. That is, the interplays between HLA class II gene family can be considered as severe specific characteristic. The interplays between the XCL family was also demonstrated as a COVID-19 severity-specific molecular interplay. In particular, the identified network comprising Human Leukocyte Antigen (HLA) class 2, CIITA, and CD74 likely played a role in COVID-19 severity, i.e., it was demonstrated that the antiviral activities of CIITA and CD74 protect against coronaviruses [21]. We also revealed that the identified severe specific gene network is enriched in "hsa04612: Antigen processing and presentation". Genes in the identified subnetwork were verified in the literature." On page 00.

Please avoid indicating the structure of the manuscript.

The comment 6) of reviewer 1 was *"6) Provide the organization of the paper at the end of introduction section"*, thus we did not delete the structure of our manuscript in the end of introduction section as follow,

"The remainder of this paper is organized as follows. In the Methods section, we propose a novel strategy for differentially regulated gene network identification based on Kullback–Leibler divergence. Then, we present the results of the simulation studies in the Monte Carlo simulation section. Finally, we describe the results of COVID-19 severity-specific molecular interplay identification. The conclusions are presented in the Discussion section." On page 3.

If you think that the structure of the manuscript should be deleted, please let us know. We will delete the part.

The authors should add the bibliographical references missing in several phrases throughout the text.

The writing includes excessive technical jargon, making it difficult for readers to follow the strategy employed and understand the implications of the findings.

In order to help readers understand better, we have added additional explanations of technical jargons and related references/URLs in overall our manuscript.

The conclusion appears vague. Could the authors delve into the novelty of their findings?

We have described the novelties of our study in the viewpoint of a computational strategy and

discoveries related to COVID-19 severity in Conclusion section as follow,

"The novelties of our study can be considered in the viewpoint of a computational strategy and discoveries related to COVID-19 severity. In the viewpoint of computational strategy: the developed computational strategy performs differentially gene network identification based on the comprehensive information of network (i.e., not only expression levels of genes but also gene network structure), and thus we can effectively perform differential gene network analysis. In the viewpoint of discovery related to COVID-19 severity: a novel discovery of our study was severe specific molecular interplays of HLA class II and CIITA, while the existing studies focused on the biomarker identification of not molecular interplays but a single gene." On pages 12-13.
