# Supplementary material for: Computational network biology analysis revealed COVID-19 severity markers: Molecular interplay between HLA-II with CIITA
Source: PLoS One. 2025 Mar 31;20(3):e0319205. doi: 10.1371/journal.pone.0319205 (PMC11957389; doi:10.1371/journal.pone.0319205)
Supplement: Attachment — Submitted filename: Response to reviewer_R2.pdf [file pone.0319205.s002.pdf]

Response to Editor on

"Computational network biology analysis revealed COVID-19 severity markers: Molecular interplay between HLA-2 with CIITA"

We would like to thank you for insightful comments and suggestions. We carefully incorporated the comments and suggestions in the revised version of our manuscript. Our point by point responses are given in the following:

In the abstract, I recommend using the acronym COVID-19 instead of repeatedly writing "coronavirus disease 2019." The same applies to the term "Human Leukocyte Antigen." They only need to be explained at the first mention in the introduction.

We have modified the abstract by using acronyms COVID-19 and HLA, and the acronyms are explained at the first mention in the Introduction section.

As in the first letter, I again suggested the authors end the introduction with the novelties of their experimental strategies and the importance of the findings

We have described novelties of our strategy and the importance of the findings as follows,

"The novelties of our study are trying to uncover COVID-19 severe specific biomarkers based on the molecular interplays not abnormalities of each single gene and developing a novel computational strategy for identifying differentially regulated gene networks between COVID-19 severe and non-severe samples. Our strategy revealed COVID-19 severe specific molecular interplays between HLA class II and CIITA/CD74, those cannot be revealed by the single gene-based existing studies. The identified severe-specific molecular interplays may provide vital clues to uncover severe COVID-19 mechanism, because the complex mechanism of severe COVID-19 involved with numerous perturbed genes in the molecular networks rather than a single abnormal gene." On page 3.

and eliminate the paragraph from lines 112-117. There is no need to specify the structure of the manuscript.

We have deleted the structure of the manuscript written from lines 112-117.

On line 83, I propose rephrasing "It can be through our results that..." to improve clarity.

We have rephrased the sentence as follows,

"Our results showed that the severe samples have relatively complex gene networks composed of larger number of genes and edges compared with those of the non-severe samples." On pages 2-3.

Moreover, I encourage the authors to succinctly convey the novelties of their studies in one concise sentence, avoiding the breakdown into multiple points.

According to your second and this comments, we have modified the paragraph describing novelties of our study as follows,

"The novelties of our study are trying to uncover COVID-19 severe specific biomarkers based on the molecular interplays not abnormalities of each single gene and developing a novel computational strategy for identifying differentially regulated gene networks between COVID-19 severe and non-severe samples." On page 3.

I also suggest avoiding statements such as "can effectively reveal the COVID-19 severity mechanisms of COVID-19" (line 104) since the authors rightly underlined the limitations of their study.

We modified such sentences as follows,

"This implies that our strategy incorporates comprehensive information on gene networks, which is, the mean of expression levels and gene network structure, and it leads to biologically reliable result for differential gene network analysis." On page 6

"The developed method incorporates comprehensive information about gene networks, which refers to not only the expression levels of genes, but also the network structure, and thus our strategy can provide informative results for identifying differentially regulated gene networks." On page 11

Could the author delve into the description of samples, clarifying what constitutes the severe and non-severe groups?

We have described the COVID-19 sample in detail as follows,

"The 473 samples were annotated with four levels of phenotype severity, i.e., ``Most severe (patients

in intensive care unit or requiring intubation and ventilation)", ``Severe (others requiring oxygen support)", ``Mild (other symptomatic patients)", and ``Asymptomatic (without COVID19 related symptoms)" [15]. The 368 samples with levels "Most severe" and "Severe" are defined as COVID-19 severe samples, and 105 samples composed of "Mild", and "Asymptomatic" levels are defined as non-severe samples." On page 9

At the end of the conclusions, the paragraph from lines 400 to 408 is somewhat convoluted and feels redundant in expressing key claims. A more straightforward presentation of these ideas would enhance readability and comprehension.

In order to clearly present novelties of our study, we have modified the paragraph as follows,

"The novelty of our study is trying to uncover COVID-19 severe specific biomarkers based on the molecular interplays, while previous studies focused on the abnormalities of each single gene. In order to achieve this, we proposed the novel computational strategy for identifying differentially regulated gene networks between COVID-19 severe and non-severe samples based on comprehensive information of gene networks (i.e., not only expression levels of genes but also network structure)." On page 11

Lastly, I noticed some errors in typing, so please amend them.

We have carefully read again and rewritten our manuscript without typos in overall.
